# Deep Learning-Based Diagnosis of Lumbar Spondylolisthesis Using X-Ray Imaging

**DOI:** 10.3390/diagnostics15162015

**Published:** 2025-08-12

**Authors:** Chunyang Xu, Yukan Wu, Beixi Bao, Xingyu Liu, Yiling Zhang, Runchao Li, Tianci Yang, Jiaguang Tang

**Affiliations:** 1 Department of Orthopedics, Beijing Tongren Hospital, Capital Medical University, Beijing 100730, China; chunyangxu2018@163.com (C.X.); wuyukan@mail.ccmu.edu.cn (Y.W.);; 2 School of Life Sciences, Tsinghua University, Beijing 100084, China; 3 Institute of Biomedical and Health Engineering (iBHE), Tsinghua Shenzhen International Graduate School, Shenzhen 518071, China; 4 Department of Biomedical Engineering, School of Medicine, Tsinghua University, Beijing 100084, China; 5 Longwood Valley Medical Technology Co., Ltd., Beijing 100730, China

**Keywords:** lumbar spondylolisthesis, deep learning, early diagnosis, detection algorithms, X-ray imaging

## Abstract

**Background**: Lumbar spondylolisthesis (LS) is a common spinal disorder characterized by the forward displacement of the vertebra. Early detection is challenging due to asymptomatic presentation in the early stages. This study develops and validates an AI-based deep learning model for the early, high-precision diagnosis of LS using lumbar X-ray images. **Methods**: A total of 3300 lateral lumbar X-ray images were collected from Beijing Tongren Hospital, and an external dataset of 1100 images was used for validation. The images were randomly divided into the training, validation, and test sets. The model uses semantic segmentation to precisely segment vertebral bodies and calculate distances between vertebrae to identify and grade LS using the Meyerding classification. Model performance was compared to other algorithms and clinical experts. **Results**: The model achieved F1 Scores of 0.92 and 0.91 on the hospital and external datasets, respectively, outperforming other methods. It showed diagnostic accuracies of 96.1% and 94.4%, exceeding the performance of physicians (90.6% and 89.3%). These results highlight the potential of AI in improving diagnostic accuracy and clinical decision-making. **Conclusions**: Our deep learning model demonstrates high accuracy and reliability in diagnosing LS, providing a valuable tool for early detection and better patient outcomes. Future work will involve expanding the dataset and validating the model in clinical settings.

## 1. Introduction

Lumbar spondylolisthesis (LS) is a common spinal disorder characterized by the forward displacement of the upper vertebra relative to the adjacent lower vertebra [1,2]. This condition can be caused by various factors, including congenital developmental abnormalities, trauma, and degenerative changes. In the early stages, LS is usually asymptomatic. However, as the condition progresses, patients may experience symptoms such as lower back pain, neurogenic intermittent claudication, and radiating pain in the lower limbs [3]. In severe cases, surgical intervention may be required [4]. Therefore, accurate diagnosis and timely treatment are crucial for patient recovery and prognosis.

Due to the lack of significant clinical symptoms in the early stages of LS, its diagnosis mainly relies on imaging tests. In clinical practice, X-ray imaging is one of the commonly used methods for diagnosing LS [5]. This technique allows for the observation of the alignment of the lumbar spine and joint spaces, enabling the assessment of the extent and type of the condition. X-ray examination is effective, fast, and cost-efficient. However, it also has certain limitations. For example, X-rays may struggle to accurately detect minor LS, and it cannot establish a direct correlation between its symptoms and clinical signs. Moreover, the subtle nature of early symptoms of LS, along with the limitations of physician subjectivity and experience during the diagnostic process, also presents challenges to traditional X-ray-based diagnosis of LS [6].

In recent years, deep learning—a subset of artificial intelligence (AI)— has made revolutionary progress across various fields, including medical imaging. Specifically, convolutional neural networks (CNNs) have demonstrated exceptional performance in image recognition tasks by automatically learning relevant features from large datasets [7,8]. These advancements suggest that deep learning can significantly improve the diagnostic accuracy and efficiency of various medical conditions, including LS. In 2022, Mohammad et al. developed a deep transfer learning model for diagnosing scoliosis and spondylolisthesis, achieving significant improvements in diagnostic accuracy [9]. Similarly, Deepika et al. developed the VGG16 and InceptionV3 models for diagnosing LS, which also yielded promising results [10]. However, despite the encouraging results from existing studies, the deep learning algorithms employed are primarily detection algorithms, which offer limited interpretability, leading to challenges in providing a clear explanation for LS diagnoses and posing potential risks for medical diagnostics.

Moreover, most of the studies conducted thus far have relatively small sample sizes, which may affect the generalizability and accuracy of the results. Furthermore, there is limited research focused on further grading and diagnosing spondylolisthesis. Therefore, we believe that applying deep learning models to the diagnosis of LS requires further in-depth research and development.

Therefore, this study aims to develop and validate a fully automated, interpretable deep learning model for the diagnosis and grading of lumbar spondylolisthesis from lateral X-ray images. By integrating semantic segmentation and traditional image analysis techniques with the clinically established Meyerding classification system, we seek to improve the accuracy and clinical utility of AI-based diagnostic tools for spinal disorders.

## 2. Materials and Methods

### 2.1. Data Collection

We collected a total of 3300 lateral lumbar X-ray images from patients who visited Beijing Tongren Hospital between January 2021 and January 2023 (hospital dataset), along with 1100 lateral lumbar X-ray images from patients at Haidian Hospital in Beijing during the same period (external dataset). The imaging data were continuously collected through the Picture Archiving and Communication System (PACS). All included patients were over 50 years old and underwent lateral lumbar X-ray examinations. Exclusion criteria included (1) a history of spinal surgery; (2) the presence of spinal tumors or tuberculosis; (3) severe scoliosis; and (4) poor image quality due to inconsistent positioning or suboptimal imaging standards. This study was approved by the Ethics Committee of Beijing Tongren Hospital.

### 2.2. Dataset

**Figure 1:**
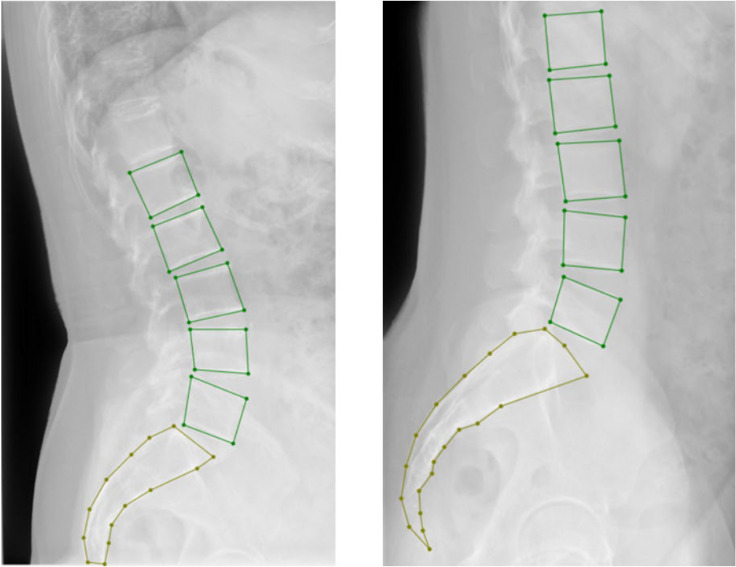
Illustration of the vertebra labeling approach.

All selected 3300 lumbar images in the hospital dataset were collected via the Digital Imaging and Communications in Medicine (DICOM) system. After converting the DICOM images into JPG format, three professional spinal specialists annotated the images using LabelMe. The annotation included (1) marking the lumbar spine from L1 to L5 with bounding boxes and (2) marking the entire sacrum with points (Figure 1). After randomizing the data, the hospital dataset was split into the training, validation, and test sets in a 7:2:1 ratio, with the 1100 images in the external dataset used as the test set (Table 1). The average age and gender composition of patients in each dataset showed no significant differences. The distribution of GR-classified lumbar spondylolisthesis (LS) grades across the datasets is presented in Table 2.

### 2.3. Model Workflow

**Figure 2:**
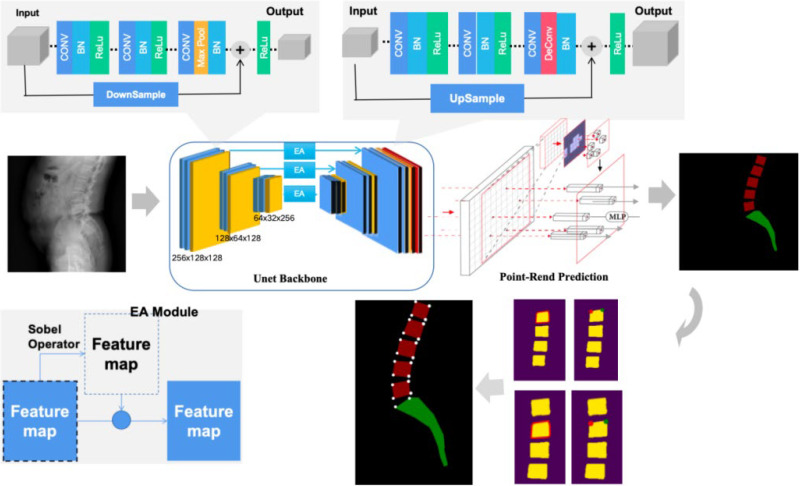
Architecture and workflow of the developed deep learning-based semantic segmentation model by introducing the PointRend Unet model.

The raw lateral lumbar X-ray dataset is processed through our proposed semantic segmentation model, which combines semantic segmentation and traditional image processing methods to extract image features and understand their semantic information. The image size is reduced to create a set of model datasets. The model is then trained, and its performance is tested on the test set. If the detection accuracy does not meet the required standards, the network is adjusted, and the training continues. Once the detection accuracy reaches the required threshold, the model is finalized (Figure 2). The output of the model, combined with the Meyerding classification, is used to determine whether the patient has spondylolisthesis. Images classified as Meyerding Grade I or higher indicate the presence of spondylolisthesis.

### 2.4. Semantic Segmentation Model with PointRend Unet

**Figure 3:**
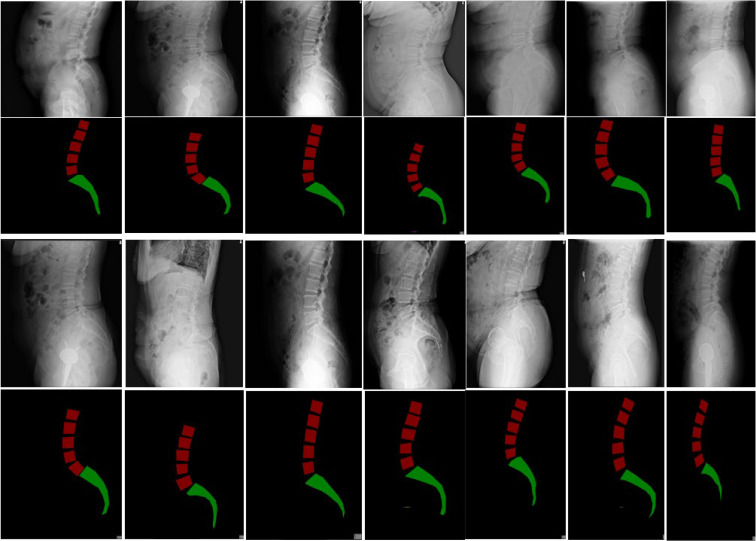
Visualization of the semantic segmentation model.

The main structure of the semantic segmentation model consists of an encoder and a decoder. The encoder’s primary role is to extract semantic features from the image. By extracting these features, the model can fully understand the semantic information in the lateral spinal X-ray images. The encoder uses 4 structurally similar blocks, each containing two 3 × 3 convolutional layers, to continually abstract the high-level semantic features of the image, reducing the image size to one-quarter of the original input size, thus lowering the computational load of the model. The decoder, which has a U-shaped structure corresponding to the encoder, is responsible for upsampling the feature maps extracted by the encoder and merging them with the corresponding feature maps from the encoder, restoring the input image’s resolution. The encoder and decoder are connected through skip connections, allowing the feature maps obtained at each stage of the encoder to be fused with those obtained in the corresponding stages of the decoder. This process helps retain high semantic information and improves the segmentation performance of the model. In the skip connection part of the model, an attention mechanism specifically designed for edge feature extraction (edge-attention), is incorporated to enhance the segmentation model’s ability to improve edge detection in the image. The visualization of the semantic segmentation model is shown in Figure 3.

### 2.5. Encoder

The encoder consists of 4 structurally similar blocks. Each block contains two 3 × 3 convolutional layers, a ReLU activation function as the activation layer, a Batch Normalization layer to accelerate model training and improve model stability, and a max-pooling layer. The max-pooling layer progressively abstracts the high-level semantic features of the image and reduces the image size to one-quarter of the input, thus decreasing the model’s computational load.

### 2.6. Decoder

The decoder is composed of 4 blocks similar in structure to those in the encoder. Each block includes two 3 × 3 convolutional layers, a ReLU activation function, a Batch Normalization layer, and a transpose convolutional layer. The transpose convolutional layer is used to upsample the image, effectively restoring its resolution.

### 2.7. Edge Attention

**Figure 4:**
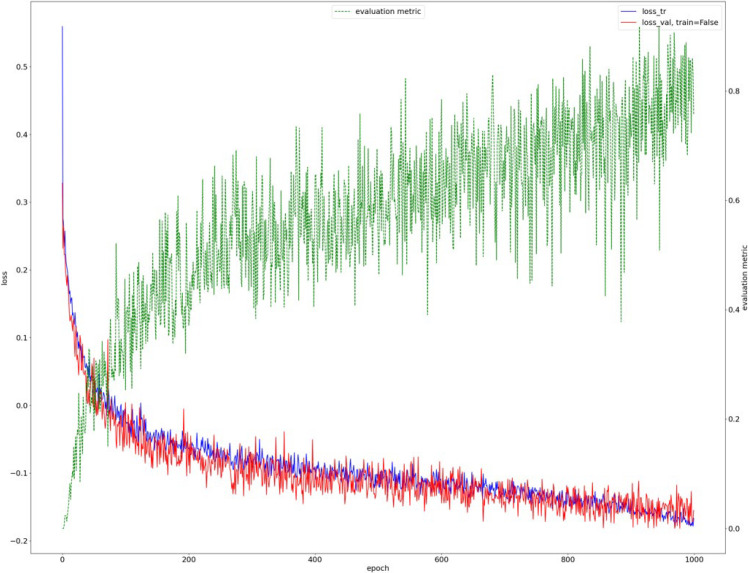
Model training loss function.

The attention mechanism is structured as shown in Figure 4. Before the feature maps extracted at each stage of the encoder are combined with those from the decoder, the edge information of the feature maps is first extracted using the Canny edge detection algorithm. This edge information is then concatenated with the original feature map image. The resulting feature map is subsequently added to the feature map from the decoder at the same stage, completing the feature fusion process through the skip connection.

### 2.8. PointRend Section

PointRend is an image segmentation algorithm designed to enhance the segmentation model’s ability to handle edges and fine details. In traditional semantic segmentation tasks, the edges of certain regions may become distorted due to operations like pooling or convolution, leading to less precise segmentation boundaries. PointRend addresses this issue by introducing localized processing, allowing the model to better capture the boundaries and finer details of the target.

### 2.9. Finding Measurement Points

After obtaining the segmented image, the mask of the lumbar spine is first filtered using labels. The mask for L1 is found through connected component analysis. Then, the convex hull and bounding rectangle of the L1 mask are calculated to find the upper-left and upper-right corners, which correspond to the upper anterior and posterior edges of L1. The lower-left and lower-right corners are then computed to represent the lower anterior and posterior edges of L1. Following a similar process, the upper anterior, upper posterior, lower anterior, and lower posterior edges for L2, L3, L4, and L5 are determined. For the sacrum’s upper anterior and upper posterior edges, as shown in Figure 2, the mask for the sacral portion is extracted, and its convex hull and bounding rectangle are computed to obtain the upper-left and upper-right intersections of the bounding rectangle and convex hull, which define the upper anterior and upper posterior edges of the sacrum (S1).

### 2.10. Data Annotation and Inter-Rater Reliability

The lumbar spine X-ray images were annotated by three spinal experts who were blinded to the clinical outcomes of the patients. The annotators independently marked the vertebral bodies from L1 to L5 and the sacrum (S1) on each image. The annotations were performed using the LabelMe tool. In the event of any disagreement between the annotators, they discussed the discrepancies and reached a consensus through collaborative discussion. This consensus-based approach ensured that the final annotations reflected the collective agreement of all annotators.

To assess the consistency and reliability of the annotations, inter-rater agreement was calculated using Fleiss’ Kappa statistic, which is commonly employed for assessing agreement among multiple raters. Fleiss’ Kappa was computed for both the vertebral body segmentation and sacrum labeling stages. The results of the inter-rater reliability analysis showed substantial agreement (Kappa = 0.02, *p* < 0.05), indicating high consistency between the annotators.

### 2.11. Training Parameter Settings

During model training, to balance training speed and stability, the learning rate is set to 0.0001. The Adam optimizer is used, with a batch size of 16 and 500 iterations. To prevent overfitting, L2 regularization is applied. The focal loss function is used as the loss function to address the imbalanced sample distribution. Additionally, an early stopping mechanism is implemented to halt training when model performance no longer improves, particularly when the loss function changes by less than 10% after 50 iterations. This helps prevent overfitting.

### 2.12. Meyerding Classification

The Meyerding classification is a common method used to describe the degree of spondylolisthesis, primarily applied to the lumbar spine. The main criterion divides the upper edge of the lower vertebra into four equal parts. Based on the degree of anterior displacement of the upper vertebra relative to the lower vertebra, the classification is divided into four grades:

Grade I: The vertebral slip does not exceed 1/4 of the sagittal diameter of the vertebra’s midsection.
Grade II: The vertebral slip exceeds 1/4 but does not exceed 2/4 of the sagittal diameter of the vertebra’s midsection.
Grade III: The vertebral slip exceeds 2/4 but does not exceed 3/4 of the sagittal diameter of the vertebra’s midsection.
Grade IV: The vertebral slip exceeds 3/4 of the sagittal diameter of the vertebra’s midsection.

### 2.13. Evaluation Metrics

We selected three metrics—Precision, Recall, and F1 Score—to assess the performance of our model:

$$Precision=\frac{TP}{TP+FP}$$

$$Recall=\frac{TP}{TP+FN}$$

$$F1\;Score=\frac{2\;*\;(Precision\;*\;Recall)\;}{\left(Precision+Recall\right)}$$

where *TP* (true positive) represents the number of samples correctly classified as the positive class, *FP* (false positive) denotes the number of negative class samples incorrectly classified as positive, and *FN* indicates the number of positive samples incorrectly classified as negative. *Precision* measures how many of the samples that the model classified as positive are truly positive. *Recall* indicates how many of the true positive samples the model was able to correctly identify. The *F1 Score* is the harmonic mean of *Precision* and *Recall*, offering a balance between the model’s accuracy and comprehensiveness.

Additionally, to compare the diagnostic performance between the deep learning model and that of clinical doctors in diagnosing LS, we established a group consisting of three clinical doctors and compared their results. The three clinicians included two senior clinicians with over five years of experience and a chief clinician (none of the clinicians participated in the diagnosis or labeling process).

## 3. Results

The model training loss function is shown in Figure 4. In this experiment, a total of 10,300 iterations were conducted, and the model began to converge after the 9700th iteration (Figure 4).

The deep learning model achieved high recognition accuracy, with an *F1 Score* of 0.92 on the in-house validation dataset and 0.91 on the external dataset. In comparison, the senior clinicians achieved *F1 Scores* of 0.90 and 0.89 on the two datasets, respectively (Table 3).

## 4. Comparison with Other Models

YOLOv7 (You Only Look Once version 7) is a deep learning model for object detection and represents the latest version in the YOLO series. It aims to improve object detection performance through enhanced model structure and training methods. One of the key features of YOLOv7 is its use of a multi-scale feature pyramid network (FPN), which helps to extract and integrate semantic information from different scales in the image. By introducing FPN, YOLOv7 improves the model’s ability to handle multi-scale objects, enhancing its detection performance for small and distant objects.

CenterNet is another deep learning model for object detection, where the core idea is to predict the center point of the object for detection. Compared to traditional object detection methods, CenterNet has made significant improvements in both accuracy and speed. It uses a novel architecture that transforms object detection into a regression problem. Unlike other methods, it predicts not only the bounding box of the object but also the position of the object’s center. This design allows the model to focus on the core information of the object, which effectively improves detection accuracy.

We trained the YOLOv7 and CenterNet models with the same iterations and compared their performance with that of our model on the test set. The results show that our model achieves higher recognition accuracy (Table 4).

### Testing of the LS Grading Model

To further compare the performance of our proposed approach in LS subtype classification, we used the Meyerding classification method and performed a comparison on the performance between the proposed algorithm and doctors on both the in-house and external datasets.

**Figure 5:**
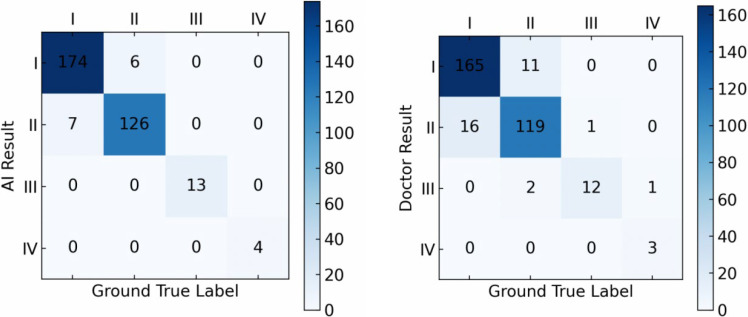
The confusion matrix comparison of AI model results and doctors’ results on the hospital dataset.

On the in-house dataset, our model achieved an accuracy of 96.1% in LS grading, while that of the doctors was 90.6% (Figure 5).

**Figure 6:**
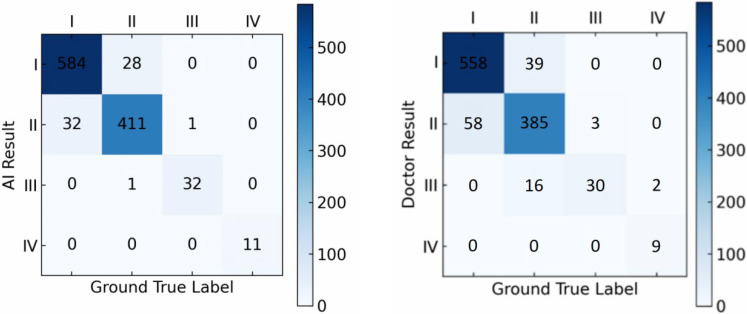
The confusion matrix comparison of AI model results and doctors’ results on the external dataset.

On the external dataset, our model achieved an accuracy of 94.4% in LS grading, while that of doctors in grading LS was 89.3% (Figure 6).

The comparisons indicate that, for both the hospital dataset and the external dataset, our proposed deep learning-based detection model outperforms orthopedic doctors in LS classification performance, especially in grading LS of types I and II, where the model performs better than the doctors.

We further applied the McNemar test to evaluate whether the accuracy differences between the model and the doctors were statistically significant. The results indicated that the performance difference between the model and the doctors was statistically significant (*p* < 0.05), further confirming the model’s superior performance.

## 5. Discussion

In this study, we introduced a fully automated and clinically applicable deep learning (AI)-powered system with the PointRend Unet model introduced to assist doctors in diagnosing LS from lumbar X-ray images. The effectiveness of our model was validated through extensive experiments on a large-scale real-world clinical dataset, which included 3,300 lumbar spine lateral X-ray images, demonstrating its superior performance. Comparison studies with the diagnostic results of experienced clinical doctors in lumbar X-ray segmentation showed that the PointRend Unet approach provides more effective analysis of the lumbar X-ray images. We also evaluated PointRend Unet model performance on an external dataset, which also achieved satisfactory results of 96.1% and 94.4% accuracies on both datasets compared to other existing deep learning-based methods.

Deep learning is crucial for developing automated and accurate methods for clinical diagnosis in digital medicine. The use of lumbar spine X-ray images for diagnosing lumbar spine diseases still remains a fundamental challenge, where the precise identification of vertebral margins has not yet been thoroughly explored and resolved. Traditional LS diagnostic methods heavily rely on the subjective judgment and experience of radiologists. Though effective, they are often time-consuming and prone to human error [11,12]. In contrast, deep learning models process X-ray images using convolutional neural networks and other advanced algorithms, providing a more automated, efficient, and accurate diagnostic approach [13,14,15]. These models can quickly analyze large volumes of highly consistent imaging data, reducing the inherent variability and bias in manual evaluations [16,17]. By improving diagnostic accuracy and enabling early detection, they significantly enhance the utilization of healthcare resources. This advancement is especially beneficial in resource-limited settings, where this technology can provide expert-level diagnostics and is expected to improve diagnostic speed and accuracy across various healthcare environments [18,19].

For example, Zhang et al. developed a new deep learning model, the Faster Region-based Convolutional Neural Network (R-CNN), which demonstrated better accuracy, Recall, and F1 Scores than those by the doctor group in LS detection. Furthermore, with the assistance of the deep learning model, the accuracy, Recall, and F1 Scores of the doctor group increased by 4.8%, 8.2%, and 6.4%, respectively, while the average diagnosis time per X-ray was reduced by 7.139 s [5]. Xuan et al. also trained a deep artificial neural network (ANN) model labeled by clinically experienced doctors to classify 604 MRI cases into normal, disk herniation, or LS. The best-performing model achieved an overall accuracy of 90% [20]. Zhao et al. proposed a new method based on the Faster Adversarial Recognition (FAR) network, which can diagnose LS in MRI images by detecting key vertebrae without the need for landmark localization. The X-ray images achieved an accuracy of 89.33% ± 2.76% [21].

Previous studies have shown that many scholars have explored using deep learning for diagnosing LS. However, most of the current studies rely on object detection techniques, which have certain limitations. First, deep learning models are often considered as “black boxes,” with their decision-making process lacking transparency. This may lead to insufficient interpretability in the field of medical diagnosis, thereby increasing potential risks. Second, while object detection methods may perform well in locating the slip position, they face challenges when grading the degree of spondylolisthesis, primarily due to the uneven distribution of samples. Specifically, data for types I and II spondylolisthesis are relatively easier to obtain, while data for types III and IV are rarer, which limited the training performance of classification models as they require a large and balanced dataset to accurately learn the features of different spondylolisthesis subtypes. For patients with type I and type II spondylolisthesis, the lumbar spine images are clearer and the segmentation effect is better, leading to better recognition results. These drawbacks suggest that traditional object detection methods may not fully meet the high demands for Precision and interpretability in clinical diagnosis.

To address the limitations of object detection techniques in diagnosing LS and to overcome the shortcomings of previous research, we developed this innovative deep learning-based approach that combines semantic segmentation algorithms and graphical algorithms by introducing the PointRend Unet model. We first use the semantic segmentation algorithm to segment the lumbar spine and sacrum and then use the graphical algorithm to identify key points on these structures and calculate distances. Finally, the Meyerding classification system is applied to diagnose and grade the degree of spondylolisthesis. Moreover, we designed an edge attention module in the proposed model to improve the accuracy of vertebral edge segmentation, which more accurately delineates the vertebral edges, improving both the diagnostic and grading accuracy. In comparison with other similar models and clinical doctors, our approach achieved higher accuracy. We also adopted the Meyerding classification system to diagnose and classify LS, thereby enhancing the interpretability of the deep learning-based diagnosis. This eliminated the issue of the lack of transparency in the deep learning model’s decision-making process, making the evaluation of our model based on a widely recognized clinical grading system.

The results show that, whether on our hospital dataset or on external hospital dataset, our model achieves high accuracy even for types III and IV spondylolisthesis, which are more structurally complex and have severe occlusions. This success is due to our algorithm and the use of the Meyerding classification system for LS detection, which helps bypass the issue of sample imbalance. This innovation is especially significant, as it enables our model to maintain high recognition accuracy for LS types that are less represented in the dataset, such as LS of types III and IV.

In the model’s accuracy evaluation, our model significantly outperforms similar models like YOLOv7 and CenterNet by achieving *F1 Scores* of 0.92 and 0.91 on the hospital and external datasets, respectively. Moreover, in the grading tests on both datasets, our model achieves accuracy scores of 96.1% and 94.4%, respectively, surpassing the accuracy scores of 90.6% and 89.3% by clinical doctors, indicating that our model has higher sensitivity and specificity in both the diagnostic and classification accuracy of LS.

## 6. Limitation

Although the proposed model achieved a high classification accuracy of 96.1% on the test set and accurately graded LS, we acknowledge certain limitations in this study. First, all data in this study were from a single region, so the sample size and diversity need to be improved to include different age groups, genders, and ethnicities in order to verify the generalizability of the model. Second, there may still be some limitations in recognizing types III and IV LS, where data is scarce. Larger and more diverse datasets are needed to further validate the model’s diagnostic and classification ability for LS. Additionally, while the edge attention module designed improved the accuracy of vertebral edge segmentation, its effectiveness may depend on specific dataset characteristics, and its performance needs to be further verified under different imaging conditions in future studies. Additionally, this study is designed as a retrospective analysis, primarily focusing on model development and validation using both internal and external datasets. Prospective clinical trials, diagnostic efficiency evaluations, and image reading assistance experiments have not yet been conducted. In the future, we will conduct a series of prospective experiments to evaluate the real-world application effects of AI-assisted diagnosis, including diagnostic time, referral decisions, and changes in treatment strategies. Finally, although our model shows potential in improving diagnostic accuracy for LS, it should currently be seen as an assistive tool for doctors rather than a replacement. When making the final diagnosis, doctors still need to consider all clinical information and apply necessary professional judgment.

## 7. Conclusions

In summary, this study presents a novel and clinically interpretable deep learning model for the diagnosis and grading of lumbar spondylolisthesis based on X-ray imaging. By combining semantic segmentation with traditional geometric measurement techniques and using the widely accepted Meyerding classification, our model achieves high accuracy, sensitivity, and specificity, surpassing both traditional object detection models and experienced clinicians in performance. These findings underscore the potential of deep learning to enhance diagnostic consistency and support clinical decision-making. In the future, further validation in larger, multi-center, and more diverse populations will be essential to facilitate clinical translation and deployment.

## Figures and Tables

**Table 1:** The sources of the research dataset.

Dataset	Training Set	Validation Set	Test Set
Hospital Dataset	2310	660	330
External Dataset	-	-	1100

**Table 2:** Grading data of lumbar spondylolisthesis.

	Grade I	Grade II	Grade III	Grade IV
Hospital Dataset	181	132	13	4
External Dataset	616	440	33	11

**Table 3:** The *Precision*, *Recall*, and *F1 Score* of our approach and those of the doctors in different datasets.

Performance	Hospital Dataset			External Dataset		
	*Precision*	*Recall*	*F1 Score*	*Precision*	*Recall*	*F1 Score*
Our Approach	0.96	0.92	0.92	0.94	0.91	0.91
Doctors	0.91	0.91	0.90	0.89	0.89	0.89

**Table 4:** Comparison of the *Precision*, *Recall,* and *F1 Score* of our approach with those of YoloV7 and CenterNet in different datasets.

Performance	Hospital Dataset			External Dataset		
	*Precision*	*Recall*	*F1 Score*	*Precision*	*Recall*	*F1 Score*
Our Approach	0.96	0.92	0.92	0.94	0.91	0.91
YoloV7	0.87	0.9	0.88	0.88	0.89	0.88
CenterNet	0.85	0.9	0.87	0.86	0.89	0.87

## Data Availability

The generated datasets in this study are not publicly available because the data were created based on patient images but are available from the corresponding author upon a reasonable request.

## Abbreviations

The following abbreviations are used in this manuscript:

LS	Lumbar Spondylolisthesis
AI	Artificial Intelligence
CNNs	Convolutional Neural Networks
YOLOv7	You Only Look Once version 7
FPN	Feature Pyramid Network
R-CNN	Region-based Convolutional Neural Network
FAR	Faster Adversarial Recognition
